# Classification-Predictive Model Based on Artificial Neural Network Validated by Histopathology and Direct Immunofluorescence for the Diagnosis of Oral Lichen Planus

**DOI:** 10.3390/diagnostics14141525

**Published:** 2024-07-15

**Authors:** Katarzyna Osipowicz, Piotr Turkowski, Izabela Zdolińska-Malinowska

**Affiliations:** 1 Department of Immunodermatology, Medical University of Warsaw, Koszykowa 82a, 02-008 Warsaw, Poland; 2 Faculty of Health Science, Calisia University, Nowy Świat 4, 62-800 Kalisz, Poland; 3 OT.CO Zdrowie Sp. z o.o., Bartycka 24B/U1, 00-716 Warsaw, Poland; 4 Escritor.pl, Jana Pawła II 27, 00-867 Warsaw, Poland

**Keywords:** artificial neural networks, artificial intelligence, predictive models, lichen planus, oral lesions

## Abstract

The diagnosis of oral lichen planus (OLP) poses many challenges due to its nonspecific clinical symptoms and histopathological features. Therefore, the diagnostic process should include a thorough clinical history, immunological tests, and histopathology. Our study aimed to enhance the diagnostic accuracy of OLP by integrating direct immunofluorescence (DIF) results with clinical data to develop a multivariate predictive model based on the Artificial Neural Network. Eighty patients were assessed using DIF for various markers (immunoglobulins of classes G, A, and M; complement 3; fibrinogen type 1 and 2) and clinical characteristics such as age, gender, and lesion location. Statistical analysis was performed using machine learning techniques in Statistica 13. The following variables were assessed: gender, age on the day of lesion onset, results of direct immunofluorescence, location of white patches, locations of erosions, treatment history, medications and dietary supplement intake, dental status, smoking status, flossing, and using mouthwash. Four statistically significant variables were selected for machine learning after the initial assessment. The final predictive model, based on neural networks, achieved 85% in the testing sample and 71% accuracy in the validation sample. Significant predictors included stress at onset, white patches under the tongue, and erosions on the mandibular gingiva. In conclusion, while the model shows promise, larger datasets and more comprehensive variables are needed to improve diagnostic accuracy for OLP, highlighting the need for further research and collaborative data collection efforts.

## 1. Introduction

Mouth ulcers, which affect around 25% of the adult population with their peak onset occurring between 10 and 29 years of age [1,2], comprise an umbrella term that can have many different causes including mechanical injuries (such as rough foods, brushing teeth, and dental prosthetics); viral, bacterial, or fungal infections [3,4,5]; genetic factors [6], mainly related to systemic inflammatory diseases (such as Crohn’s disease [7], systemic lupus erythematosus [8], and Behçet’s disease [9]); locally acting irritating chemical agents (such as using whitening toothpaste, excessive alcohol consumption, and smoking) [10]; and allergic/adverse reactions to food, drugs [11], or dental materials. Lichen planus is also one of the possible causes of this problem. This mucocutaneous condition affects the oral cavity. The disease is prevalent in 1% of the population [12], being most commonly observed in females aged between 30 and 70 years. To diagnose suspected ulcerative lichen planus, a biopsy is essential due to the 0.44–2.28% risk of malignant lesion occurrence [13]. In lichen, a dense subepithelial lymphocytic band is observed on hematoxylin–eosin staining. The epithelium is keratotic with basilar degeneration and the presence of Civatte bodies (degenerating keratinocytes). A sawtooth appearance of the rete ridges may be present. However, lesions do not always yield a specific image on standard microscopy, so lichen cannot always be unequivocally confirmed or excluded on histopathological examination. Lesions that resemble lichen are called lichenoid lesions.

Oral lichen planus is a chronic inflammatory and autoimmune disorder. Although the exact cause of OLP is still not fully understood, it is thought to result from a disturbance in cell-mediated immune function due to a combination of genetic and environmental factors [14]. While indirect immunofluorescence and immunohistochemistry are not useful in OLP diagnosis [15,16], direct immunofluorescence (DIF) is considered a helpful tool for the differential diagnosis of oral lichen planus and oral lichenoid lesions [17]. Direct immunofluorescence is primarily used to diagnose blistering diseases such as pemphigus and pemphigoids in erosive lesions, particularly desquamative gingivitis [18]. In lichen planus, a shaggy deposition of fibrinogen and complement is observed along the basement membrane zone, with no immunoglobulin present other than in colloid bodies [15]. However, fibrinogen deposition is not specific to OLP as other oral potentially malignant disorders (OPMDs) can exhibit a similar pattern [15]. Moreover, deposits of fibrin at the basement membrane zone and IgM-positive cytoid bodies similar to those in OLP may be present also in oral lichenoid drug reactions [15].

In the absence of new diagnostic tools, algorithms designed to shorten the diagnostic process are particularly important. Diagnostic criteria for OLP were published in 1978 by the WHO; in 2003, they were modified by van der Meij and van der Waal [19], and in 2016, by the American Academy of Oral and Maxillofacial Pathology. However, these official guidelines do not fulfill informational needs; for these reasons, various authors have undertaken the creation of diagnostic schemes. In 2019, Bilodeau and Lalla introduced a diagnostic algorithm for oral lesions, which was based solely on clinical symptoms [20]. The following year, Holla et al. [21] published a diagnostic algorithm for oral mucosal blistering diseases derived from histopathological and immunological findings but did not incorporate clinical symptoms. Also in 2019, Rashid et al. [22] published a diagnostic pathway flowchart for patients with oral blistering, focusing on mucous membrane pemphigoid, pemphigus vulgaris, and paraneoplastic pemphigus. Another 2019 publication [23] was characterized by a cautious approach, avoiding definitive statements and merely indicating the likelihood of specific diagnoses without providing numerical estimates. This publication advised referring patients to specialized hospitals and considering various diagnostic tests. The conservative nature of this tool, while reflective of the authors’ careful methodology, limits its usefulness for clinical decision making.

We are witnessing an explosion of interest in the application of artificial intelligence (AI) in many fields including medicine. Machine learning (ML), a branch of AI, focuses on making predictions by identifying patterns within data. A specialized subset of ML, deep learning, uses multilayered neural network algorithms modeled after the human brain’s complex structure to make predictions. ML processes training data to identify distinctive characteristics in medical records or images, subsequently classifying them into various disease categories. Neural network learning can be either supervised, where correct answers are provided, or unsupervised, where the network clusters objects based on their similarities. The performance reliability of ML is evaluated by validating these acquired features with separate validation data and further confirming through testing with a dedicated dataset [24]. In this article, as in the Statistica 13 software (Polish version), we refer to completing training as ‘testing’ and the assessment of classification accuracy as ‘validation’, which is the opposite of the usual practice in the literature of the field.

In our view, none of the diagrams published until now were sufficiently comprehensive to effectively support clinicians. Therefore, we intended to combine medical history and DIF to create a new multivariate predictive model. For this purpose, we used a semi-finished product such as Statistica as none of the authors are programmers. However, the involvement of an AI specialist comes at a very high cost, so the purpose of this work was to make a preliminary screening assessment, using an off-the-shelf tool, of whether the integration of history, histological, and immunological data using a neural network is a promising avenue for the further development of AI-based diagnostic tools for the diagnosis of lichen.

## 2. Materials and Methods

### 2.1. Basic Statistics

Statistical analysis was performed using Statistica 13. Qualitative variables were presented as numbers and percentages; for the continuous variable ‘age’, the median, interquartile range, and range were given. The relationship between the assessed qualitative variables was assessed using the chi-square method, taking 0.05 as the level of significance. The continuous variable ‘age’ between groups was compared using the Mann–Whitney U test.

### 2.2. Variants of Classification

Confirmation of lichen via histopathology is very difficult. Many results are false-negative in histopathology despite clinical signs indicative of lichen. All patients who had a biopsy had clinical symptoms indicative of lichen as suspected lichen was an inclusion criterion. According to diagnostic standards, lichen can be diagnosed if the histopathology result does not exclude such a diagnosis and the clinical picture is consistent with the disease. Thus, we conducted the analysis in two variants. In the first interpretation, a very narrow one, only patients with lichen confirmed in HP were classified as having lichen. In the second interpretation, standard for clinical practice, lichen was present in all patients in whom it was not excluded. Neural networks were created only for the variable “lichen not excluded in histopathology”.

### 2.3. Artificial Neural Networks

Automatic neural networks were used as an alternative to multivariate analysis as it was not possible to perform logistic regression due to the anomalies of the maximum likelihood estimator in this dataset. Due to the redundancy of variables relative to cases, the Data Mining module of Statistica was used to select an appropriate number of variables (in a ratio of 1:10 relative to cases, i.e., 8 variables) that could be entered into the machine learning model in which predictors’ significance was calculated by ranking the *p*-values for each predictor effect (for related *p*-values, the rankings were based on the ranking of the F). The number of confirmed cases of lichen in the study sample prevented the use of machine learning methods to find predictors for the variable lichen confirmed (lichen features on histopathology) as this would have risked overfitting the model after splitting it into a learning, test, and validation sample. Instead, such an analysis was performed for lichen features present or not excluded in histopathology. We used the default parameters of the Statistica 13 software (200 epochs; random, Gaussian initialization; stopping condition: 0.0000001; multilayer perceptron; cross validation). The following variables were assessed: gender, age on the day of lesion onset, DIF IgG, DIF IgA, DIF IgM, DIF C3, DIF F1, DIF F2, lip lesions, nail lesions, stress during the study period, stress at onset, genital symptoms, erosions on palate, erosions on buccal mucosa (right side/left side), erosions on tongue, erosions under tongue, erosions on maxillary gingiva, erosions on mandibular gingiva, erosions on upper lip, erosions on lower lip, white patches on palate, erosions on buccal mucosa (right side/left side), white patches on tongue, white patches under tongue, white patches on maxillary gingiva, white patches on mandibular gingiva, white patches on lower lip, white patches on upper lip, whether patient was previously treated by a dermatologist, whether patient was previously treated by a dentist, whether patient was previously treated by a general practitioner, any previous treatment, taking supplements, taking herbs, taking any medication, dental status, smoking, flossing, and using mouthwash. Four statistically significant variables were selected for machine learning.

## 3. Results

### 3.1. Study Population

The study group consisted of 80 patients: 63 (78.8%) women and 17 (21.2%) men. Lichen was confirmed by histopathology in four (5.0%) of the study participants and not confirmed in fifty-seven (71.2%); it was not excluded in thirty subjects (37.5%) and excluded in thirty-one (38.8%). The remaining values represent missing data (non-diagnostic histopathology). The characteristics of the patients participating in the study are shown in Table 1.

### 3.2. Direct Immunofluorescence vs. Histopathology

The incidences of DIF IgG, DIF IgA, DIF IgM, DIF C3, DIF F1, and DIF F2 positivity did not differ significantly between either subjects with confirmed or unconfirmed lichen or between subjects with lichen excluded or not excluded (Table 2). DIF IgG, DIF IgM, and DIF IgA did not show associations with DIF F1 or DIF F2 scores.

Immunological patterns are shown in Table 3. Most of the patients (*n* = 37, 61.7%) were negative for all tested markers. Among four patients with lichen confirmed, all were negative, whereas among fifty-six patients with lichen not definitely confirmed, only thirty-three (58.9%) were negative. Among twenty-nine patients with lichen not excluded, fifteen (51.7%) were negative, and fourteen (48.3%) had another pattern, whereas among thirty-one patients with lichen excluded, twenty-two (70.1%) were negative and nine (29%) had another pattern. However, these differences were not significant (*p* = 103 and *p* = 126, respectively). The immunological pattern was not available for two patients.

There was a correlation between DIF F1 and DIF F2 scores and DIF C3 scores (Table 4). In the overall group, a positive DIF F1 score increased the probability of a positive DIF C3+ score by four times (OR 4.080, 95% CI: 1.0909 to 15.2594, *p* = 0.0367) while a positive DIF F2 score increased this probability by almost five times (OR 4.892, 95% CI: 1.2902 to 18.5514, *p* = 0.0196).

### 3.3. Artificial Neural Network

The variables suggested by the Data Mining module as being relatively best for creating a predictive model are shown in Table 5.

Machine learning resulted in five networks with very similar parameters (Table 6).

**Figure 1:**
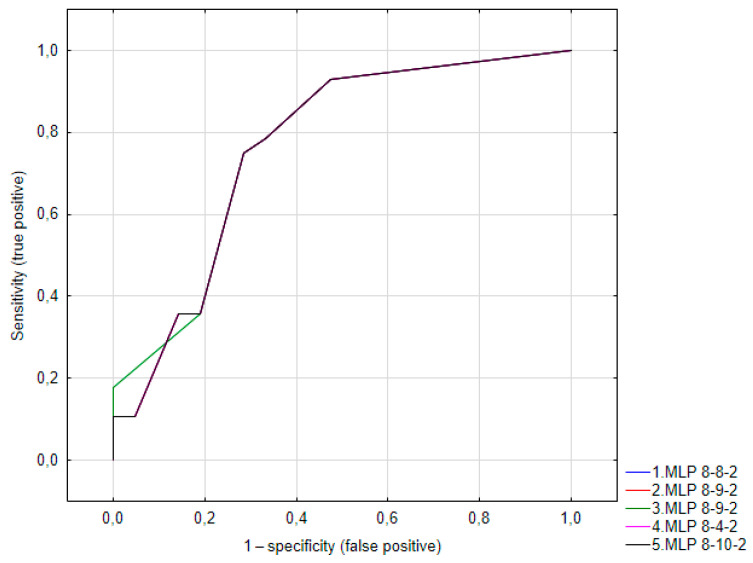
ROC curve or variable “lichen not excluded in histopathology”. MLP: multilayer perceptron. The number represents the number of neurons in the input layer, in the hidden layer, and in the output layer.

The models created in this way achieved approximately 71% correct classifications in the validation sample. The ROC curves for the models are shown in Figure 1.

## 4. Discussion

Our study confirmed previous observations regarding the weak association between the localization of lesions and the diagnosis of lichen. In this group of patients, erosions or white patches under the tongue excluded this diagnosis, and erosions on the mandibular gingiva significantly decreased the risk of having a lichen lesion but did not exclude it. Meanwhile, in the study conducted by Keller, 41.6% of 79 patients with OLP had lesions on the gingiva; for the rest of the cases, the gingival lesions were connected to other areas, particularly the buccal mucosa and tongue [25]. Another study described lesions diagnosed as OLP mainly on the buccal mucosa (93.9%) followed by the gingiva (59.7%), mucobuccal fold (26.8%), tongue (26.8%), palate (7.3%), and vermilion border (7.3%) [18]. These differences suggest that the localization of lesions is significant but still unclear and cannot be an independent criterion for diagnosis.

In our study, stress was a significant predictor for lesions identified as ‘lichen not excluded’. However, we did not confirm the significance of this variable for lichen confirmed in HP. The association between stress and oral lesions has been well described in the literature. De Porras-Carrique et al. [26] conducted a meta-analysis that included 51 studies involving 6815 patients. It showed that patients with oral lichen planus (OLP) had a high prevalence of depression (31.19%), anxiety (54.76%), and stress (41.10%). Compared to the control group without OLP, OLP patients had significantly higher rates of depression (OR  =  6.15, 95% CI  =  2.73–13.89, *p*  <  0.001), anxiety (OR  =  3.51, 95% CI  =  2.10–5.85, *p*  <  0.001), and stress (OR  =  3.64, 95% CI  =  1.48–8.94, *p*  =  0.005), with large effect sizes. However, this factor is not specific to lichen planus. Stressful situations can trigger a transitory increase of salivary cortisol and stimulate immunoregulatory activity by increasing the number of leukocytes and natural killer cells in inflammatory sites, as well as the production of cytokines and antibodies, which are commonly observed during the development of mouth ulcers [27,28,29,30]. A study by Huling et al. [27] involving 160 recurrent aphthous stomatitis patients showed that the odds of experiencing a recurrent aphthous stomatitis episode increased almost threefold (odds ratio [OR] = 2.72; 95% confidence interval [CI] = 2.04–3.62) when exposed to a stressful life event. When compared to physical stressors, psychological stressors (such as an examination or job interview) had a greater effect on the occurrence of recurrent aphthous stomatitis episodes (OR = 3.46, 95% CI = 2.54–4.72). Wang et al. [31] also described a strong link between stressful life events and recurrent aphthous stomatitis. They analyzed summary statistics from the largest publicly available GWAS on Europeans, which included data from 461,106 individuals with mouth ulcers and 10 psychiatric traits: anxiety disorder (*n* = 83,566), attention-deficit/hyperactivity disorder (*n* = 53,293), autism spectrum disorder (*n* = 46,350), bipolar disorder (*n* = 51,710), insomnia (*n* = 1,331,010), major depressive disorder (*n* = 480,359), mood instability (*n* = 363,705), neuroticism (*n* = 168,105), schizophrenia (*n* = 105,318), and subjective wellbeing (*n* = 388,538). Their analysis revealed that autism spectrum disorder, insomnia, major depressive disorder, and subjective wellbeing had significant effects on mouth ulcers, with corresponding odds ratios (ORs) of 1.160 (95% confidence interval [CI]: 1.066–1.261, P = 5.39 × 10^−4^), 1.092 (1.062–1.122, P = 3.37 × 10^−10^), 1.234 (1.134–1.342, P = 1.03 × 10^−6^), and 0.703 (0.571–0.865, P = 8.97 × 10^−4^), respectively. They also found some suggestive evidence that mood instability may cause mouth ulcers, with an instrumental variable-weighted (IVW) OR of 1.662 (1.059–2.609, P = 0.027).

In the diagnosis of lichen planus, DIF is used to detect the presence of anti-keratinocyte antibodies in the patient’s tissue. These antibodies are directed against keratin antigens, which are found on the surfaces of keratinocytes and the basement membrane of the skin. Positive DIF in patients with lichen has been described repeatedly but rates have varied. For example, the percentage of patients found to have fibrinogen deposits was 37–100% [32,33]. The variation in the percentage of positive DIF results may be due to the different clinical patterns of lichen, of which there are six: reticular, papular, plaque-like, atrophic, erosive, and bullous. In one study, fibrinogen deposition was found at the BMZ in 73% of cases of reticular OLP and 57% of cases of plaque-like OLP [28]. It may also depend on the location of the lesion. Bujeeb, who investigated 85 Thai patients with OLP, observed positive DIF in 94% of biopsies taken from the buccal mucosa, 64% of those taken from the gingiva, and 50% taken from the palate [18]. In 2022, Mao et al. [33] described eight patterns of DIF in patients with OLP. Among 65 patients, 15 (35.7%) were quadruple (IgM, IgA, IgG, C3)-negative; two (4.8%) were quadruple-positive; ten (23.8%) were positive only for C3; seven (16.7%) were positive only for IgM, four (9.5%) were positive for IgM and IgA, three (7.2%) were positive for IgM and C3 and one (2.4%) was positive for IgM, IgG, and C3. Additionally, the two clinical subtypes did not differ from each other in terms of DIF. Similar analysis conducted by Bujeeb, including the location of deposits, revealed 12 DIF patterns, of which the most common were the following: a) fibrinogen deposits along the basement membrane zone (F BMZ) and IgM at colloid bodies (CBs) (35%); b) F BMZ (28%); c) F BMZ and IgM and IgA at CBs (12%); and d) F BMZ and IgM, IgA, and C3 at C (10%). Other types were sporadic (1.5% each).

In our study, none of the four patients with OLP confirmed in HP had a positive DIF result. By substituting the values known from the literature as the probability of a random event into a binomial distribution, we obtained, from 0.16, a success probability of 37%, and from 0.0016, a success probability of 80%. This shows that the percentage we obtained was significantly different from the results of at least those authors who obtained the highest percentages, but the size of only four people in this group does not allow us to draw radical conclusions.

The studies by other authors cited above, as well as our results, showed the limitations of using DIF for the diagnosis of lichen, probably due to the complex nature of this disease, having multiple phenotypes. Our attempt to create a multivariate classification model did not allow us to create a diagnostically useful tool based on the dataset we collected. Neural networks created based on our dataset achieved approximately 71% correct classifications in the validation sample. This value is too low to recommend the creation of a clinically useful tool on this basis but suggests that further work on optimizing the model on a larger number of cases and new variables acquired (in particular after including more patients with histopathologically confirmed lichen, which would allow the dependent variable to be changed) could potentially lead to such a tool. Perhaps the combination of data collected by several authors would allow the creation of such a tool. It should be borne in mind, however, that the neural networks that would be applied in clinical practice meet the criteria of a medical device and are subject to all the resulting regulations. Despite the difficulties involved in the development and eventual registration of effective neural networks, their use could help resolve OLP classification and diagnosis problems, but for this, it is necessary to find better predictors than those collected in our study. In particular, they may be useful when there are serious contraindications to biopsy.

Our study had several limitations. First, there were only four patients with lichen confirmed in HP. Given that there are six types of lichen, our sample did not reflect all phenotypes of this disease, especially as patients were not stratified by this type. Unlike the authors cited above, we did not collect detailed data on the location and appearance of deposits within the cell, which would have provided another variable that could have been included in the analysis. Furthermore, in many cases, biopsies were not performed or the results were non-diagnostic, which narrowed the dataset for analysis. Although histopathology is a gold standard, this examination can also yield unreliable results. Last, we used commercially available statistical software. The information provided by the software manufacturer regarding methods and results is limited and does not contain the full data that would be available if the network were created directly by a developer. Ideally, further work on the development of tools should involve an artificial intelligence specialist. This report can only serve as a preliminary screening analysis, indicating the possibility and necessity of further work in this direction.

Nevertheless, studies of this type are scarce in the literature, so despite these limitations, our work has made some contribution to the current state of knowledge by at least expanding the pool of patients described in this way. Furthermore, our analysis complements the 2022 publication by Mao et al. [33], whose authors assessed the correlation of IgG, IgM, IgA, C3, and C4 with each other and with RAE score and flow cytometry results but did not include fibrinogen.

Due to all the above limitations, our publication is very preliminary. The main takeaway from this work should be that such classification tools based on neural networks, increasingly used for decision making in medicine and veterinary science [34], can be particularly useful in clinical problems like the diagnosis of lichen planus. However, it is necessary to develop such tools using larger datasets than those we have gathered. We are open to collaborating with other researchers who possess similar data or can collect them.

The optimal direction for the further development of AI-based tools for OLP diagnosis seems to be the creation of models that integrate data from history, immunological tests, and imaging studies. Neural networks have shown high accuracy in classifying superficial images of lesions as OLP vs. non-OLP (88.18%) [35]. In the research conducted by Keser et al. [36], a network trained on photographic images of the buccal mucosa, including 65 healthy samples and 72 samples with oral lichen planus lesions, achieved 100% accurate classifications, confirmed by experts in Oral Medicine and Maxillofacial Radiology. These results are very encouraging but the test and validation sample sizes in this study were very small (*n* = 7), resulting in a high risk of overfitting. Not only an increase in sample size but also the inclusion of ultrasound images could improve the quality of classification. The ultrasonography of oral mucosal lesions is a relatively new method that faces many barriers. The miniaturization of probes without the loss of image quality is a technical challenge; the probes used in the available literature are prototypes or instruments adapted from other areas of medicine [37]. A systematic review published in 2023 showed that most of the few publications on the ultrasound of oral lesions concerned animals or healthy participants [37]. There is currently only one publication on the use of ultra-high-frequency ultrasound in patients with OLP [38]. Its authors presented ultrasound findings but, due to the small sample size, did not determine the sensitivity or specificity of this test in this indication. A similar paper by the same authors in patients with pemphigus vulgaris (PV) or mucous membrane pemphigoid (MMP) showed 75% sensitivity in the diagnosis of PV and 66.7% in the diagnosis of MMP [39]. As can be seen, no diagnostic tool singly guarantees reliability in the diagnosis of OLP, so it is necessary to continue the development of multivariate classification models that integrate imaging, clinical, and immunological data. Ultimately, however, it must be underlined that any classification model that would enter clinical practice must be registered as a medical device in the EU [40]; such registration may be required also in the USA [41].

## 5. Conclusions

In conclusion, we confirmed that OLP poses diagnostic difficulties and that factors described in the literature as being associated with OLP are not univariate predictors. The use of an advanced method of analyzing the collected variables resulted in quite good classification, but the quality of this model is still insufficient for the use of such a tool in clinical practice, so the search for further diagnostic features is indispensable.

## Figures and Tables

**Table 1:** Characteristics of the study group based on the relative best predictors for the dependent variable analyzed (lichen not excluded in HP).

	Lichen Confirmed in HP			LP Not Excluded in HP		
	No (*n* = 57)	Yes (*n* = 4)	*p*-Value	No (*n* = 30)	Yes (*n* = 30)	*p*-Value
Sex Female Male	46 (93.9) 11 (91.7)	3 (6.1) 1 (8.3)	0.781	25 (51.0) 6 (50.0)	24 (49.0) 6 (50.0)	0.949
Age at onset Median (IQR)	60 (40–64)	59 (52–64)	0.751	62 (41–65)	60 (43–64)	1.0
Stress at onset No Yes	25 (96.1) 22 (88.0)	1 (3.9) 3 (12.0)	0.279	19 (73.1) 10 (40.0)	7 (26.9) 15 (60.0)	0.017
Patient previously treated by a GP No Yes	41 (91.1) 4 (100.0)	4 (8.9) 0 (0.0)	0.533	23 (51.1) 4 (100.0)	22 (48.9) 0 (0.0)	0.059
White patches under the tongue No Yes	43 (91.5) 5 (100.0)	4 (8.5) 0 (0.0)	0.497	23 (48.9) 5 (100.0)	24 (51.1) 0 (0.0)	0.029
White patches on buccal mucosa No Yes	12 (100.0) 36 (90.0)	0 (0.0) 4 (10.0)	0.254	9 (75.0) 19 (47.5)	3 (25.0) 21 (52.5)	0.094
Erosions on mandibular gingiva No Yes	31 (91.2) 19 (95.0)	3 (8.8) 1 (5.0)	0.604	14 (41.2) 14 (70.0)	20 (58.8) 6 (30.0)	0.041
Erosions under the tongue No Yes	45 (91.8) 4 (100.0)	4 (8.2) 0 (0.0)	0.552	24 (49.0) 4 (100.0)	25 (51.0) 0 (0.0)	0.049

*p*-values marked with red are statistically significant (*p* < 0.05).

**Table 2:** DIF results in patient subgroups distinguished by histopathology.

N = 63	Lichen Confirmed on HP		*p*-Value	Lichen not Excluded on HP		*p*-Value
	No (*n* = 57)	Yes (*n* = 4)		No (*n* = 33)	Yes (*n* = 30)	
DIF IgG (−)	53 (93.0)	4 (7.0)	0.635	29 (50.9)	28 (49.1)	0.594
DIF IgG +	3 (100.0)	0 (0.0)		2 (66.7)	1 (33.3)	
DIF IgA (−)	55 (93.2)	4 (6.8)	0.787	31 (52.5)	28 (47.5)	0.297
DIF IgA (+)	1 (100.0)	0 (0.0)		0 (0.0)	1 (100.0)	
DIF IgM (−)	53 (93.0)	4 (7.0)	0.635	29 (50.9)	28 (49.1)	0.594
DIF IgM (+)	3 (100.0)	0 (0.0)		2 (66.7)	1 (33.3)	
DIF C3 (−)	45 (91.8)	4 (8.2)	0.327	25 (51.0)	24 (49.0)	0.832
DIF C3 (+)	11 (100.0)	0 (0.0)		6 (54.6)	5 (45.4)	
DIF F1 (−)	40 (90.9)	4 (9.1)	0.212	25 (56.8)	19 (43.2)	0.185
DIF F1 (+)	16 (100.0)	0 (0.0)		6 (37.5)	10 (62.5)	
DIF F2 (−)	42 (91.3)	4 (8.7)	0.253	26 (56.5)	20 (43.5)	0.173
DIF F2 (+)	14 (100.0)	0 (0.0)		20 (35.7)	9 (64.3)	

**Table 3:** Immunological patterns. + means positive test result, − means negative test result.

IgG	IgA	IgM	C3	F1	F2	N (% among OLP Confirmed)	N (% among OLP Not Confirmed)	N (% among OLP Not Excluded)	N (% among OLP Excluded)
−	−	−	−	−	−	4 (100.0)	33 (58.9)	15 (51.7)	22 (71.0)
−	−	−	+	+	+	0 (0)	6 (10.8)	3 (10.3)	3 (9.7)
−	−	−	−	+	−	0 (0)	2 (3.6)	1 (3.4)	1 (3.2)
−	−	−	−	+	+	0 (0)	7 (12.5)	6 (20.7)	1 (3.2)
+	−	+	+	−	−	0 (0)	1 (1.8)	0 (0)	1 (3.2)
−	−	+	−	+	+	0 (0)	1 (1.8)	0 (0)	1 (3.2)
+	+	−	−	−	−	0 (0)	1 (1.8)	1 (3.5)	0 (0)
−	−	−	+	−	−	0 (0)	3 (5.7)	2 (6.9)	1 (3.2)
+	−	−	+	−	−	0 (0)	1 (1.8)	0 (0)	1 (3.2)
−	−	+	−	−	−	0 (0)	1 (1.8)	1 (3.5)	0 (0)

**Table 4:** Relationship between DIF IgG, DIF IgM, DIF IgA, and DIF C3 results and DIF F1 and DIF F2 results.

	DIF F1 (−)	DIF F1 (+)	*p*-Value	DIF F2 (−)	DIF F2 (+)	*p*-Value
DIF IgG (+)						
No	52 (71.2)	21 (28.8)	0.208	54 (74.0)	19 (26.0)	0.240
Yes	4 (100.0)	0 (0.0)		4 (100.0)	0 (0.0)	
DIF IgA (+)						
No	54 (72.0)	21 (28.0)	0.840	56 (74.7)	19 (25.3)	0.756
Yes	2 (66.7)	1 (33.3)		2 (66.7)	1 (33.3)	
DIF IgM (+)						
No	54 (73.0)	20 (27.0)	0.320	56 (75.7)	18 (24.3)	0.252
Yes	2 (50.0)	2 (50.0)		2 (50.0)	2 (50.0)	
DIF C3 (+)						
No	51 (77.3)	15 (22.7)	0.028	53 (80.3)	13 (19.7)	0.013
Yes	5 (45.5)	6 (54.5)		5 (45.5)	6 (54.5)	

*p*-values marked with red are statistically significant (*p* < 0.05).

**Table 5:** Variables proposed to be entered into the analysis by the Data Mining module for the variable ‘lichen planus not excluded by histopathology’. For lichen confirmed, all variables were not significant.

Variable	Chi Value	*p*-Value
Age at onset	6.219780	0.622628
Stress at onset	5.684772	0.017113
White patches under the tongue	4.741641	0.029441
Erosions on mandibular gingiva	4.190498	0.040651
Erosions under the tongue	3.862974	0.049363
Patient previously treated by a GP	3.548971	0.059582
White patches on buccal mucosa (left side)	2.808929	0.093741

*p*-values marked with red are statistically significant (*p* < 0.05).

**Table 6:** Automatic neural network classifiers for the variable ‘lichen not excluded in HP’.

Network ID	Quality (Learning)	Quality (Testing)	Quality (Validation)	Learning Algorithm	Function Error	Activation (Hidden)	Activation (Output)
MLP 8-8-2	74.28571	85.71429	71.42857	BFGS 4	Entropy	Linear	Softmax
MLP 8-9-2	74.28571	85.71429	71.42857	BFGS 4	SOS	Logistic	Linear
MLP 8-9-2	74.28571	85.71429	71.42857	BFGS 2	Entropy	Linear	Softmax
MLP 8-4-2	74.28571	85.71429	71.42857	BFGS 3	Entropy	Linear	Softmax
MLP 8-10-2	74.28571	85.71429	71.42857	BFGS 2	SOS	Tanh	Logistic

MLP: multilayer perceptron. The code in the first column represents the number of neurons in the input layer—the number of neurons in the hidden layer—and the number of neurons in the output layer.

## Data Availability

The dataset is available on request from the authors.
